# Case Report: Congenital diaphragmatic hernia associated with esophageal atresia and tracheoesophageal fistula

**DOI:** 10.3389/fsurg.2022.1061951

**Published:** 2023-01-04

**Authors:** Feihong Zhang, Yang Wu, Li Zhang, Bin Xia

**Affiliations:** ^1^ Department of Pediatrics, West China Second Hospital, Sichuan University, Chengdu, China; ^2^ Key Laboratory of Birth Defects and Related Diseases of Women and Children, Sichuan University, Ministry of Education, Chengdu, China

**Keywords:** congenital abnormalities, diaphragm defect, deformities, premature infants, surgery

## Abstract

The coexistence of congenital diaphragmatic hernia and esophageal atresia with or without tracheoesophageal fistula is extremely rare; only 36 cases have been reported. We report a case of a preterm male newborn infant with left congenital diaphragmatic hernia, esophageal atresia, and tracheoesophageal fistula and review 27 related cases.

## Introduction

Congenital diaphragmatic hernia (CDH) is a developmental defect in the diaphragm that occurs in approximately 1 in 3,300 live births (1). A defect in the diaphragm causes herniation of the abdominal organs into the chest and compression of the lungs, which results in respiratory insufficiency after birth and persistent pulmonary hypertension of the newborn (2). Esophageal atresia (EA) and tracheoesophageal fistula (TEF) are some of the most common congenital abnormalities of the gastrointestinal tract, affecting 1 in 3,000–4,500 live births (3). The co-occurrence of CDH and EA with TEF is very rare and carries a high fatality rate of 51.9%. The fatality rates of right and left CDH and EA with TEF were estimated to be 60% and 44.4%, respectively, while that of left CDH with EA was 80%. Surgery is the only method to correct these deformities. Cases of early postoperative survival have been reported since 1993. However, there is no unified method to treat these two deformities. We report a case of a premature infant with left CDH, EA, TEF and a right aortic arch.

## Case report

A male infant weighing 2,350 g with an antenatal diagnosis of left CDH and right aortic arch since the 24-week gestation period was born by cesarean section at 35^+2^ weeks of gestation. No abnormality was found in amniotic fluid low density gene chip at 21 weeks of gestation. The left CDH was revealed by systematic fetal ultrasound and targeted ultrasound at 24 weeks of gestation. The size of the right lung in the cross section was about 2.4 × 1.57 cm and the lung-to-head ratio (LHR) was about 1.64. Follow-up targeted fetal echocardiography and fetal chest MRI suggested right aortic arch. Fetal ultrasound at 26 weeks of gestation showed polyhydramnios (amniotic fluid: 8.4 cm, amniotic fluid index: 26.0 cm). Targeted ultrasound at 34 weeks of pregnancy showed that the lung-to-head ratio was 1.93.

The baby had mild respiratory distress at birth, with Apgar scores of 5 and 8 at 1 and 5 min, respectively. Immediately after birth, the infant was intubated and admitted to the neonatal intensive care unit (NICU). Attempts to pass a nasogastric tube (NGT) were unsuccessful, and EA was suspected. Chest radiography confirmed the presence of left CDH with multiple air-filled loops of the bowel in the thoracic cavity, which suggested the possibility of EA and TEF with a U-shaped NGT in the middle mediastinum. Emergency surgery was performed after the evaluation. The spleen, stomach, and most of the intestines were returned to the abdominal cavity *via* a transverse incision of the left upper abdomen, and a 3 × 3 cm defect of the left posterolateral diaphragm was observed(Defect B according to CDH Study Group Staging System). The diaphragmatic defect was intermittently sutured using a non-absorbable suture. It was difficult to maintain the infant's breathing under high-frequency ventilation (HFV); therefore, we did not attempt to perform a thoracotomy to ligate the fistula. A gastrostomy was performed to prevent gastrointestinal flatulence caused by mechanical ventilation. After the first operation, the infant suffered severe pulmonary hypertension and underwent treatment with high-frequency oscillatory ventilation, nitric oxide inhalation, and cetuximab. His blood oxygen saturation under HFV was normal. Subsequently, pulmonary hemorrhage occurred, and a hemodynamically significant patent ductus arteriosus (HsPDA) was considered. Ibuprofen was used for the closure of the PDA. After these treatments, the patient underwent thoracotomy *via* the left posterolateral incision on day of life (DOL) 16. Due to a large amount of pleural effusion in the pleural cavity and evident mediastinal edema, only the TEF was ligated and divided. Esophageal anastomosis was performed on DOL 39; the distance between the proximal and distal ends was 2.5 cm.

The patient was fed through a gastrostomy tube on DOL 20. No signs of esophageal stricture or gastroesophageal reflux were found on esophagography 10 days after esophageal anastomosis. Unfortunately, the patient developed intestinal failure-associated liver disease due to long-term parenteral nutrition. Biochemical indices, such as liver enzymes and serum conjugated bilirubin, returned to normal after treatment with fish oil monotherapy. The patient was on oral total enteral feeding when he was discharged from the hospital at the age of 3 months. On follow-up at the age of 6 months, the patient was able to raise his head steadily and turn his body over.

## Discussion

To the best of our knowledge, 36 cases of CDH, EA, and TEF have been reported to date. We included a total of 27 cases with detailed information (Table 1) in this literature review. There were 17 cases of left CDH, EA, and TEF; 5 cases of left CDH and EA; and 5 cases of right CDH, EA, and TEF. No cases of right CDH or EA have been reported to date. Most reported cases were accompanied by other malformations, the most common of which was a cardiovascular malformation (22.2%), such as a patent ductus arteriosus, atrial septal defect, interruption of the inferior vena cava, right aortic arch, or common arterial trunk. Other malformations included lung agenesis (18.5%), Meckel's diverticulum (14.8%), renal dysplasia (11.1%), trisomy 18 (7.4%), spinal deformities (7.4%), and facial deformities (3.7%). CDH, EA, and TEF are fatal deformities in children; however, surgery has been the key to saving their lives since 1970 (4).

**Table 1:** Summary of findings of CDH associated with EA or EA and TEF.

Author	Year	Gestational age	Sex	Side of CDH	Type of EA (based on gross classification)	Associated anomalies
Ahmed (4)	1970	?	Male	Right	III	Meckel's diverticulum
Adelman (5)	1976	?	?	Left	Associated with TEF	–
Gibon (6)	1978	?	?	Left	II	Left lung agenesis
Bowen (7)	1983	38 weeks	Female	Left	II	18-trisomy, left lung agenesis
Rawlings (8)	1984	33 weeks	Male	Right	II	Patent ductus arteriosus
Udassin (9)	1987	36 weeks	Male	Left	III	Left lung agenesis, Meckel's diverticulum
	1987	35 weeks	Female	Left	I	Lung agenesis
Takehara (10)	1993	36 4/7 weeks	Male	Left	I	–
Bösenberg (11)	1994	?	Male	Left	I	–
	1994	?	Male	Left	Associated with TEF	Vertebral anomalies, choanal atresia, right renal agenesis
Nowicky (12)	1995	?	Male	Left	Associated with TEF	–
Sapin (13)	1996	34 weeks	Male	Left	III	–
Al-Salem (14)	1997	33 weeks	Male	Left	I	–
Thakral (15)	1998	Term birth	Male	Right	III	Meckel's diverticulum
Zhao (16)	2000	42 weeks	Female	Left	III	-
Cunat (17)	2005	35 6/7 weeks	Male	Left	III	Truncus arteriosus communis
Bagci (18)	2009	33 6/7 weeks	Male	Left	III	Meckel's diverticulum
Are (19)	2010	33 6/7 weeks	Male	Left	I	–
Abdul (20)	2013	36 weeks	Male	Left	III	Bilateral cryptorchidism
Charles (21)	2014	34 weeks	Male	Left	III	Patent ductus arteriosus
Evans (22)	2014	30 weeks	Female	Right	Associated with TEF	Interrupted inferior vena cava, patent ductus arteriosus, atrial septal defect
Zahn (23)	2015	38 weeks	Female	Right	III	18-trisomy, multiple cardiac malformations
	2015	36 weeks	Female	Left	III	Lung agenesis
	2015	31 weeks	Female	Left	III	VACTERL association (vertebral anomalies, anal atresia, cardiac malformation, tracheoesophageal fistula or esophageal atresia, renal anomalies, limb malformations)
	2015	33 weeks	Female	Left	III	–
	2015	35 weeks	Male	Left	III	–
	2015	32 weeks	Female	Left	III	–
Current case	2021	35 2/7 weeks	Male	Left	III	Right aortic arch, patent ductus arteriosus

CDH, congenital diaphragmatic hernia; EA, esophageal atresia; TEF, tracheoesophageal fistula.

### Treatment of left CDH, EA, and TEF

Left CDH with EA and TEF is the most common combination of deformities, and our review includes a total of 17 cases with this combination (Table 1). Among them, 13 of the cases had relevant surgical records (Table 2). In 8 cases of staged surgeries, CDH was repaired through an abdominal incision followed by esophageal anastomosis through a right thoracotomy, and a total of 4 patients survived. On average, the diaphragmatic hernia was repaired on DOL 1, and the TEF and EA were repaired between DOL 10 and DOL 14. The shortest discharge time was DOL 48, and the longest was 6.5 months. One of the patients that survived underwent diaphragmatic hernia repair on DOL 1, ligation and division of the TEF on DOL 13, and repair of the EA on DOL 49, with a total discharge time of 4 months after birth. Our patient also underwent three surgeries and was discharged at the age of 3 months. The repair of EA and TEF was performed on DOL 1 in only one reported cases (5). He was discharged at the age of 4 months and started to develop normally at 10 months old (5). In addition, two patients underwent a one-stage operation (13, 23). One died on DOL 113 due to chronic pulmonary insufficiency. The other was discharged at the age of 3 months. In general, both one-stage surgery and staged surgery seem to be reasonable choices. One-stage operation is taken into consideration only if the patient's condition permits. Interestingly, although most surgeons believed that the repair of diaphragmatic hernia should be put in the first place in staged surgery, repairing the EA first does not seem to have much to do with the survival rate(5). Therefore, we believed that the first stage of surgery should be more individualized according to the patient's condition.

**Table 2:** Summary of treatments of left CDH associated with EA or EA and TEF.

Author	Operative Procedures	Recommended treatment strategy	Outcome
Bowen (7)	–	Four ways to improve ventilation: (1) gastrostomy; (2) pass a catheter through trachea (Endotracheal intubation) and TEF into stomach; (3) inflate the balloon of Fogarty to occlude the fistula; (4) selectively intubate and ventilate.	Died before operation
Udassin (9)	Diaphragmatic hernia repair, ligation and division of TEF and gastrostomy *via* abdominal incision	Ligate and separate TEF through abdominal incision.	Died shortly after operation
BÖsenberg (11)	Diaphragmatic hernia repair and gastrostomy *via* transverse abdominal incision, occlusion of TEF with Fogarty catheter *via* gastrostomy. Five days later, repair of EA, ligation and division of TEF *via* right thoracotomy.	Selective right intubation	Alive
Nowicky (12)	Diaphragmatic hernia repair and gastrostomy *via* transverse abdominal incision on DOL 1; ligation and division of TEF *via* right thoracotomy on DOL 7	Selective right intubation	Died 6 weeks after discharge
Sapin (13)	Diaphragmatic hernia repair and gastrostomy *via* left abdominal incision, temporary circular ligation of gastroesophageal junction on DOL 1; ligation and division of TEF *via* right thoracotomy on DOL 13; repair of EA on DOL 49.	Temporarily ligate the gastroesophageal junction before ligation and division of TEF. ECOM are recommended both in thoracotomy and initial treatment.	Still alive at the age of 6.5 months
Cunát (17)	One-stage operation 6 h after birth: ligation and division of TEF and repair of EA *via* right thoracotomy, diaphragmatic hernia repair *via* left abdominal incision	Early and simultaneous surgical treatment of CDH complicated with TEF and EA has a positive effect on prognosis.	Died of chronic pulmonary insufficiency on DOL 113.
Bagci (18)	Diaphragmatic hernia repair, gastrostomy and ligation of the lower part of TEF *via* transverse abdominal incision on DOL 1; division of TEF and repair of EA *via* right thoracotomy on DOL 12.	It is feasible to ligate and divide the lower TEF *via* transabdominal transverse incision in diaphragmatic hernia repair, and the repair of EA should be delayed until the period of pulmonary hypertension had passed.	Discharged at 6.5 months
Abdul (20)	Division of TEF and repair of EA *via* right thoracotomy 6 h after birth, diaphragmatic hernia repair *via* abdominal incision on DOL 7.	A staged approach with ligation of TOF and repair of OA first followed by the delayed repair of the CDH are recommended.	Still alive at the age of 10 months
Charles (21)	Diaphragmatic hernia repair, gastrostomy and ligation of the gastroesophageal junction *via* abdominal transverse incision on DOL 1; ligation and division of TEF and repair of EA *via* right thoracotomy on DOL 12.	When the thoracic space is severely occupied due to CDH, it can be fatal to ligate TEF first without dealing with CDH.	Discharged on DOL 48
Zahn (23)	Ligation and division of TEF *via* abdominal longitudinal incision.	–	Died 48 h after birth
	Diaphragmatic hernia repair, jejunostomy, ligation and division of TEF *via* abdominal longitudinal incision on DOL 3, repair of EA *via* right thoracotomy on DOL 35.	–	Died at the age of 4 years due to pulmonary hypertension.
	Diaphragmatic hernia repair, gastrostomy and ligation of TEF *via* abdominal longitudinal incision on DOL 2, repair of EA *via* right thoracotomy on DOL 28.	–	Died at the age of 13 months
	Diaphragmatic hernia repair, ligation and division of TEF *via* longitudinal abdominal incision on DOL 2, repair of EA *via* right thoracotomy on DOL 56.	–	Still alive at the age of 8 years
	One-stage operation: displacement of the organs into an extracorporeal silastic bag *via* a longitudinal abdominal incision, ligation and division of TEF and repair of EA *via* right thoracotomy, diaphragmatic hernia repair *via* longitudinal abdominal incision.	In the case of right thoracotomy without ECMO, it is recommended to cut through the middle of the abdomen and place the abdominal organs of the left half of the chest in a silicone bag.	Discharged at the age of 3 months
Present case	Diaphragmatic hernia repair, gastrostomy *via* transverse abdominal incision on DOL 1, ligation and division of TEF *via* right thoracotomy on DOL 16, repair of EA *via* right thoracotomy on DOL 39.	It is also feasible to choose left posterolateral thoracic incision with right aortic arch.	Discharged at the age of 3 months

CDH, congenital diaphragmatic hernia; EA, esophageal atresia; TEF, tracheoesophageal fistula; DOL, day of life.

Sapin et al. (13) believed that gastrostomy was the most effective way to solve progressive flatulence caused by TEF in the gastrointestinal tract. Almost all surgical patients underwent a gastrostomy. Early gastrostomy stabilized the respiratory state and created conditions for subsequent EA and TEF repair. In three reported cases, in addition to gastrostomy, circular ligation was performed at the gastroesophageal junction to reduce air leakage caused by TEF (13, 21, 23). Among these cases, one patient reported by Zahn et al. (23) had an obvious esophageal stricture at the ligation site. Therefore, circular ligation of the gastroesophageal junction should be performed cautiously. Notably, in three cases of staged surgery, the TEF was repaired in the first stage of the operation (23). A longitudinal abdominal incision made it possible to ligate and divide the TEF in the operation of diaphragmatic hernia. Early closure of the fistula improves ventilation function by preventing gas from leaking into the intestines through the trachea, and this may have the same effect as a gastrostomy. Therefore, we recommend that TEF be repaired in the first stage of the operation as much as possible.

### Treatment of right CDH, EA, and TEF

Similar to surgical procedures for a left CDH, the routine operation for patients with right CDH with EA and TEF is transabdominal repair of diaphragmatic hernia, ligation, and separation of the TEF and EA repair *via* a right thoracic incision. Of the five reported cases of right CDH with EA and TEF, four patients underwent surgery (Table 3). Two patients died shortly after diaphragmatic hernia repair, ligation, and division of TEF. The other two patients who underwent one-stage surgery survived during follow-up. In summary, one-stage surgery seems to be a reasonable choice for patients with right-sided CDH complicated by EA and TEF.

**Table 3:** Summary of treatments of right CDH associated with EA or EA and TEF.

Author	Operative Procedures	Recommended treatment strategy	Outcome
Ahmed (4)	Ligation and division of TEF and diaphragmatic hernia repair *via* a right thoracoabdominal incision.	–	Died 30 h after operation
Rawlings (8)	Diaphragmatic hernia repair and gastrostomy *via* transverse abdominal incision, division of TEF *via* right thoracotomy.	The gastric tube was passed through the endotracheal tube. This temporary procedure can improve ventilation support before surgery but cannot prevent severe hypoxic brain injury.	Died on DOL 20
Thakral (15)	Diaphragmatic hernia repair and gastrostomy *via* transverse abdominal incision, division of TEF and repair of EA *via* right thoracotomy.	Early operation is an important reason for the survival of these kinds of patients, and the results of simultaneous correction of the two deformities and staging surgery may be the same.	Discharged at the age of 2 weeks
Evans (22)	Diaphragmatic hernia repair and gastrostomy *via* transverse abdominal incision, division of TEF and repair of EA *via* right thoracotomy.	–	Still alive at the age of 2 years
Zahn (23)	Died before surgery	–	Die within 24 h of birth

CDH, congenital diaphragmatic hernia; EA, esophageal atresia; TEF, tracheoesophageal fistula; DOL, day of life.

### Treatment of left CDH and EA

Among the five patients with CDH and EA, two died within 24–48 h due to severe persistent hypoxemia after diaphragmatic hernia repair (with or without esophagogastric anastomosis) (Table 4). Two patients underwent diaphragmatic hernia repair on DOL 3 and esophageal anastomosis on DOL 10 or 15. Although all patients died of septicemia at an advanced stage, some surgeons still believe that diaphragmatic hernia repair can be performed initially, and EA repair can be delayed while pulmonary hypertension resolves (9, 14). Only one patient survived during follow-up. In this case, EA with a low esophageal end was confirmed before surgery; therefore, a chevron incision was used to correct both deformities at the same time. Three months after discharge, the patient was still alive. Owing to the small number of cases, there is no unified surgical method for addressing combined deformities.

**Table 4:** Summary of treatments of left CDH associated with EA.

Author	Operative Procedures	Recommended treatment strategy	Outcome
Udassin (9)	Diaphragmatic hernia repair and gastrostomy *via* transverse abdominal incision.	Emergency EA repair is not necessary for patients without TEF and can be delayed until the after pulmonary hypertension resolves.	Died 24 h after operation due to severe persistent hypoxemia
Takehara (10)	Diaphragmatic hernia repair on DOL 3, repair of EA on DOL 15	–	Died of septicemia and aspiration pneumonia on DOL 161
BÖsenberg (11)	Diaphragmatic hernia repair, gastrostomy, and repair of EA.	–	Died 48 h after operation
Al-Salem (14)	Temporary repair of diaphragm defect with transabdominal peritoneal flap and gastrostomy *via* abdominal incision on DOL 3, diaphragmatic hernia repair on DOL 10.	–	Died of septicemia on DOL 27
Are (19)	Diaphragmatic hernia repair, esophagogastric anastomosis and jejunostomy *via* a chevron incision.	The chevron incision can be used to correct both CDH and low EA.	Follow-up upto 3 months after discharge

CDH, congenital diaphragmatic hernia; EA, esophageal atresia; TEF, tracheoesophageal fistula; DOL, day of life.

## Conclusion

For patients with a left or right CDH with EA and TEF, a one-stage surgery is possible when the patient's breathing is stable. In staged surgery, in addition to gastrostomy, early ligation and division of the TEF are also important for stabilizing the respiratory state of patients. In patients with left CDH with EA, some surgeons recommend staged surgery to repair the EA after correcting for pulmonary hypertension, while others use a chevron incision to correct both deformities in a one-stage surgery.

## Data Availability

The original contributions presented in the study are included in the article/Supplementary Material, further inquiries can be directed to the corresponding author/s.

## References

[1] Canadian Congenital Diaphragmatic Hernia Collaborative, Puligandla PS Skarsgard ED Offringa M Adatia I Baird R Diagnosis and management of congenital diaphragmatic hernia: a clinical practice guideline. CMAJ. (2018) 190:E103–12. 10.1503/cmaj.170206 29378870 PMC5790558

[2] Gien J Palmer C Liechty K Kinsella JP. Early abnormalities in gas exchange in infants with congenital diaphragmatic hernia. J Pediatr. (2022) 243:188–92. 10.1016/j.jpeds.2021.12.009 34929245

[3] Way C Wayne C Grandpierre V Harrison BJ Travis N Nasr A. Thoracoscopy vs. thoracotomy for the repair of esophageal atresia and tracheoesophageal fistula: a systematic review and meta-analysis. Pediatr Surg Int. (2019) 35:1167–84. 10.1007/s00383-019-04527-9 31359222

[4] Ahmed S. Right-sided Bochdalek hernia associated with esophageal atresia and tracheo-esophageal fistula. J Pediatr Surg. (1970) 5:256. 10.1016/0022-3468(70)90284-8 5419074

[5] Haium AAA Sim SW Ong LY Rajadurai VS. Congenital diaphragmatic hernia associated with oesophageal atresia and tracheooesophageal fistula in a low birth weight infant. BMJ Case Rep. (2013) 2013:bcr2013200014. 10.1136/bcr-2013-200014 23964045 PMC3761652

[6] Sapin E Berg A Raynaud P Lapeyre G Seringe R Helardot PG. Coexisting left congenital diaphragmatic hernia and esophageal atresia with tracheoesophageal fistula: successful management in a premature neonate. J Pediatr Surg. (1996) 31:989–91. 10.1016/s0022-3468(96)90432-7 8811578

[7] Zahn KB Scherf S Schaible T Wessel LM Hagl CI. Single-staged surgical approach in congenital diaphragmatic hernia associated with esophageal atresia. J Pediatr Surg. (2015) 50:1418–24. 10.1016/j.jpedsurg.2015.04.015 25962843

[8] Charles EJ Judge JM Vergales BD Randall AH Kane BJ McGahren ED Managing concomitant congenital diaphragmatic hernia, esophageal atresia, and tracheoesophageal fistula: a case report of a premature infant that achieved survival. J Pediatr Surg Case Rep. (2014) 2:239–42. 10.1016/j.epsc.2014.04.009

[9] Udassin RZOP Zamir O Peleg O Lernau OZ. Coexisting left diaphragmatic hernia and esophageal atresia. Pediatr Surg Int. (1987) 2:301–3. 10.1007/BF00176204

[10] Al-Salem AH Qaisruddin S Varma KK. Concurrent left congenital diaphragmatic hernia and esophageal atresia: case report and review of the literature. J Pediatr Surg. (1997) 32:772–4. 10.1016/s0022-3468(97)90032-4 9165477

[11] Adelman S Benson CD. Bochdalek hernias in infants: factors determining mortality. J Pediatr Surg. (1976) 11:569–73. 10.1016/s0022-3468(76)80015-2 987182

[12] Gibon Y Borde J Mitrofanoff P Lefort J. [Association of left diaphragmatic hernia, lung agenesia and esophageal atresia (author’s transl)]. Chir Pediatr. (1978) 19:261–7. 737826

[13] Bowen A. The ventilatory dilemma of coexisting diaphragmatic hernia, esophageal atresia, and tracheoesophageal fistula. Crit Care Med. (1983) 11:390–1. 10.1097/00003246-198305000-00017 6839792

[14] Rawlings JS Shetler PL Fill WL Cathcart CF. Concurrent right diaphragmatic hernia and Type C Tracheoesophogeal fistula. A case report. Clin Pediatr. (1984) 23:518–20. 10.1177/000992288402300917 6380876

[15] Takehara H Komi N Okada A Nishi M Masamune K. Left diaphragmatic hernia associated with lower esophageal atresia. Pediatr Surg Int. (1993) 8:339–40. 10.1007/BF00173360

[16] Bösenberg AT Mickel RE Hadley GP. Management of oesophageal atresia in association with congenital diaphragmatic hernia. Pediatr Anesth. (1994) 4:263–6. 10.1111/j.1460-9592.1994.tb00176.x

[17] Nowicky DJ. Simultaneous Bochdalek hernia and type C tracheoesophageal fistula. J S C Med Assoc. (1995) 91:303–4. 7658682

[18] Thakral CL Sajwani MJ. Concurrent right diaphragmatic hernia and esophageal atresia. Pediatr Surg Int. (1998) 14:96–7. 10.1007/s003830050448 9880710

[19] Meijian Zhao QMWL. Congenital esophageal atresia and tracheoesophageal fistula complicated with congenital diaphragmatic hernia: a case report. Tianjin Med. (2001) 1:61.

[20] Cunát V Stranák Z Pýcha K Tláskal T Melichar J Miletín J Congenital diaphragmatic hernia associated with esophageal atresia, tracheoesophageal fistula, and truncus arteriosus in a premature newborn. Pediatr Surg Int. (2005) 21:684–6. 10.1007/s00383-005-1443-4 15933889

[21] Bagci S Müller A Heydweiller A Bachour H Petersen C Heep A Temporary banding of a lower tracheoesophageal fistula in an infant with left congenital diaphragmatic hernia and esophageal atresia. Eur J Pediatr Surg. (2009) 19:260–3. 10.1055/s-2008-1038960 19224439

[22] Are NK Nagarjuna K Kannaiyan L. A rare association of congenital diaphragmatic hernia with lower esophageal atresia and perforation. Int J Pediatr. (2010) 2010:738546. 10.1155/2010/738546 20706657 PMC2913854

[23] Evans WN Kogut K Acherman RJ. Preserving the azygos vein when repairing esophageal atresia and tracheoesophageal fistula accompanied by interrupted inferior vena cava. Pediatr Surg Int. (2014) 30:345–7. 10.1007/s00383-013-3422-5 24122074

